# IGF2BP1 accelerates the aerobic glycolysis to boost its immune escape in hepatocellular carcinoma microenvironment

**DOI:** 10.3389/fimmu.2024.1480834

**Published:** 2024-11-13

**Authors:** Xuxing Ye, Junmei Lin, Yanping Chen, Xiaobo Wang

**Affiliations:** ^1^ Department of Traditional Chinese Medicine, Jinhua Municipal Central Hospital, Jinhua, China; ^2^ Department of Gastroenterology, Jinhua Municipal Central Hospital, Jinhua, China; ^3^ The Fourth School of Clinical Medicine, Zhejiang Chinese Medical University, Hangzhou, China

**Keywords:** N 6 -methyladenosine, hepatocellular carcinoma, PD-L1, immune escape, aerobic glycolysis

## Abstract

**Introduction:**

Energy metabolism abnormity emerges as a crucial factor that facilitates tumorigenesis by accelerating aerobic glycolysis. However, the function of N^6^-methyladenosine (m^6^A) on hepatocellular carcinoma (HCC) aerobic glycolysis and immune escape is still unclear. Here, this investigation was intended to elucidate the regulation of m^6^A ‘reader’ IGF2BP1 involved in HCC aerobic glycolysis and immune escape.

**Methods:**

The aerobic glycolysis was tested by glucose uptake, lactate, ATP generation and ECAR. The CD8^+^ T cell-mediated killing effect was tested by cytotoxicity, IFN-γ and granzyme B. The molecular interaction was confirmed by luciferase reporter assay, immunoprecipitation assay and chromatin immunoprecipitation (ChIP)-PCR.

**Results:**

Elevated IGF2BP1 expression was associated with poor prognosis in HCC patients. Functionally, IGF2BP1 emerged as an oncogenic factor that accelerated HCC aerobic glycolysis (glucose uptake, lactate, ATP generation and ECAR) and oxaliplatin resistance. Meanwhile, IGF2BP1 repressed the activated CD8^+^ T cell-mediated killing effect (cytotoxicity, IFN-γ and granzyme B) and apoptosis of HCC cells, indicating a suppressed cytotoxic T-cell response. By recognizing and binding to the m^6^A-modified sites on c-Myc mRNA, IGF2BP1 enhanced the stability of c-Myc mRNA, consequently upregulating c-Myc expression. In addition, transcription factor c-Myc targeted the programmed death ligand 1 (PD-L1) promoter region to strengthen its transcription.

**Discussion:**

Taken together, this study illustrates IGF2BP1 as a potential therapeutic target in HCC, aiming to disrupt the interplay between aberrant metabolism and immune escape.

## 1 Introduction

Hepatocellular carcinoma (HCC), also known as liver cancer, is a highly aggressive malignancy that originates from hepatocytes, the primary cells of the liver (1). It is associated with high mortality rates globally. The overall survival rate for HCC remains low, primarily due to late-stage diagnosis and limited treatment options. Early detection plays a vital role in improving survival rates, as it allows for timely intervention (2). Treatment modalities for HCC include surgical resection, liver transplantation, locoregional therapies, and systemic therapies (3). Surgical resection is considered the preferred treatment for early-stage HCC, while liver transplantation offers the best long-term survival for patients with cirrhosis (4). Ongoing research is focused on developing innovative antitumor immunity therapeutic strategies to enhance HCC outcomes.

N^6^-methyladenosine (m^6^A) RNA methylation is a prevalent and dynamic modification that plays a crucial role in various biological processes, including gene expression regulation and RNA metabolism (5, 6). Recent studies have also highlighted the involvement of m^6^A methylation in the development and progression of HCC. In HCC, m^6^A methylation has been shown to affect tumor initiation, progression, and metastasis. For instance, METTL3 (7) and KIAA1429 (8) participate in the HCC development. Therefore, m^6^A RNA methylation has emerged as a critical regulator in the pathogenesis of HCC. For the m^6^A methylation reader, emerging evidence has reported the broad and critical role on HCC. For instance, IGF2BP1 impairs nasopharyngeal carcinoma cells’ sensitivity to Taxol (9). IGF2BP1 mediated the stability of NRF2 mRNA to reduce the antitumor immunity and ferroptosis of gastric cancer (10). However, the role of IGF2BP1 on HCC is still unclear and worth exploring.

Metabolism and tumor immune response are importantly and intrinsically connected, with their precise mechanisms still to be elucidated. Recent advancements in onco-immunometabolism have elucidated the pivotal role of aerobic glycolysis (also known as Warburg effect) in the orchestration of immune escape within the tumor microenvironment (TME) (11). Aerobic glycolysis, a metabolic reprogramming phenomenon common in cancer cells, is characterized by increased glucose uptake and lactate production, even in the presence of oxygen. This metabolic shift not only fuels rapid tumor growth but also creates an immunosuppressive milieu that facilitates tumor immune escape (12). Lv et al. (2023) revealed that the Apolipoprotein L3 (APOL3) could bind LDHA and promote its ubiquitylation-related degradation and APOL3-LDHA axis can facilitate the CD8^+^ T cells cytotoxic ability through decreasing lactate concentration (13). Thus, the accumulation of lactate produced by aerobic glycolysis in the TME could inhibit the cytotoxic T lymphocyte function, contributing to the evasion of immune surveillance.

Collectively, these studies suggest that targeting the metabolic dependencies of tumor cells may offer an approach to disrupt the complex interplay between aerobic glycolysis and immune escape, potentially enhancing the efficacy of immunotherapeutic strategies. These findings observed that IGF2BP1 reduced the killing effect of CD8^+^ T cells to HCC cells in co-cultured system and accelerated the aerobic glycolysis, further suggesting that IGF2BP1 may contribute to the immune escape by modulating the HCC tumor microenvironment. This finding not only advances our understanding of HCC immunobiology but also provides a potential therapeutic target for the development of more effective treatments for HCC from the m^6^A modification pathway.

## 2 Materials and methods

### 2.1 Human normal and HCC samples

This study was approved by the ethics committee of Jinhua Municipal Central Hospital in accordance with the Declaration of Helsinki. 50 cases of HCC samples and corresponding normal tissue case specimens (**Table 1**) were collected in the Jinhua Municipal Central Hospital from 2020 to 2023. These samples were diagnosed by histopathological and clinical methods. The clinical features information for all recruited patients were collected and shown in **Supplementary Table S1**.

**Table 1:** The relationship between IGF2BP1 and clinicopathological characteristics of HCC patients.

	Total	IGF2BP1^High^ (n=25)	IGF2BP1^Low^ (n=25)	*p*
Age				
≥60	29	15	14	0.774
<60	21	10	11	
Gender				
Male	30	16	14	0.773
Female	20	9	11	
Tumor size				
<5 cm	21	9	12	0.390
≥5 cm	29	16	13	
Liver Cirrhosis				
Positive	28	15	13	0.568
Negative	22	10	12	
TNM				
I/II	24	17	7	0.004
III/IV	26	8	18	

### 2.2 Cell lines, culture and transfection

Human liver cancer cell lines (Huh7, MHCC97H) and normal human hepatic cells (THLE-3) were provided from Chinese Academy Medical Sciences (Beijing, China). HCC cells were cultured in RPMI-1640 medium supplemented with 10% fetal bovine serum (Gibco) in a 5% CO_2_ environment at 37°C. shRNA and overexpression constructs targeting IGF2BP1 were provided by GeneChem Biotech (Shanghai, China; http://genechem.bioon.com.cn/) and transfection process was performed based on manufacturer’s protocols. The shRNAs were listed in **Supplementary Table S1**.

### 2.3 RT-PCR

Total RNA from tissue samples was extracted using the RNeasy Mini Kit (Qiagen Inc., Redwood City, CA)) following the manufacturer’s protocol. The concentration and purity of RNA were determined using a NanoDrop 2000 spectrophotometer (Thermo Fisher Scientific). Complementary DNA (cDNA) synthesis was performed with 1 µg of total RNA using the SuperScript IV First-Strand Synthesis System (Invitrogen). Quantitative real-time PCR (RT-PCR) was conducted on a StepOnePlus Real-Time PCR System (Applied Biosystems) utilizing PowerUp SYBR Green Master Mix (Life Technologies). Gene-specific primers were designed using Primer-BLAST and synthesized by Integrated DNA Technologies. The thermal cycling conditions comprised an initial denaturation at 95°C for 2 minutes, followed by 40 cycles of 95°C for 15 seconds, and 60°C for 1 minute. Relative gene expression was calculated using the 2^-ΔΔCT^ method with GAPDH as the reference gene.

### 2.4 Western blot analysis

The protein levels were conducted to investigate protein expression levels. HCC cells were lysed using RIPA buffer (Thermo Fisher Scientific) supplemented with a protease and phosphatase inhibitor cocktail (Sigma-Aldrich, St. Louis, MO, USA, cat. P8340). Protein concentrations were determined by the BCA Protein Assay Kit (Pierce Biotechnology). Equal amounts of protein (30 µg) were separated by SDS-PAGE on a 10% polyacrylamide gel electrophoresis (SDS-PAGE) (Bio-Rad Laboratories) and transferred onto PVDF membranes (MilliporeSigma). Membranes were blocked with 5% non-fat milk in TBST and incubated with primary antibodies overnight at 4°C. The primary antibodies used were anti-IGF2BP1 (Cell Signaling Technology, cat. #64143), anti-c-Myc (Abcam, cat. #ab32072), anti-PD-L1 (Cell Signaling Technology, cat. #13684) and anti-β-actin (Cell Signaling Technology, cat #4970). Membranes were then washed and incubated with HRP-conjugated secondary antibodies (Jackson ImmunoResearch Laboratories). Bands were visualized using an ECL detection system (Amersham Biosciences) on a ChemiDoc MP Imaging System (Bio-Rad Laboratories).

### 2.5 Oxaliplatin sensitivity assay

Approximately 1×10^4^ cells were plated in each well of a 96-well plate. Following a 24-hour incubation period, the HCC cells underwent treatment with varying concentrations of OXA (0, 0.5, 1, 2, 4, 8, 16, 32 μg/mL). Each well then received 10ul of CCK-8 solution (Beyotime Institute of Biotechnology, Shanghai, China), and the optical density (OD) was recorded at a wavelength of 450 nm. Cells that were not exposed to any medication served as the control group (indicating 100% survival), which was utilized to determine the IC_50_ values for each chemotherapeutic agent.

### 2.6 Glucose uptake, lactate production and ATP assay

Following the transfection procedure, HCC cells were incubated in a glucose-deprived culture medium for 16 hours before being transferred to a culture medium with high glucose content under normoxic conditions for another 24 hours. Both the supernatants from the HCC cell culture medium and the HCC cells themselves were gathered. Measurements for glucose consumption, lactate output and ATP were conducted using a glucose assay kit (BioVision, Milpitas, California, USA) and a lactate assay kit (BioVision) and ATP Determination Kit (Thermo Fisher Scientific, Cat. No: A22066) in accordance with the protocols provided by the manufacturer.

### 2.7 Measurement of extracellular acidification rate

The analysis of ECAR was performed with XF96 Bioenergetic Analyzers from Seahorse Bioscience. Preparation of all compounds and media adhered to the instructions provided by the manufacturers. The procedure for ECAR analysis involved a stepwise injection of glucose (10 mM), oligomycin (1 mM), and 2-deoxyglucose (50 mM). ECAR values were adjusted for the total protein content and are expressed in mpH/min.

### 2.8 Flow cytometry

To assess apoptosis, HCC cells underwent two washes with cold PBS before being resuspended in 100 μl of 1× Binding Buffer. Subsequently, 5 μl of FITC Annexin V and 5 μl of propidium iodide (PI) were incorporated for staining purposes, which lasted 15 minutes at ambient temperature and was conducted in the absence of light. The detection of apoptosis was carried out using the FITC Annexin V Apoptosis Detection Kit I (BD Pharmagen) in strict accordance with the guidelines provided by the manufacturer. For the surface PD-L1 expression measurement, HCC cells underwent staining for 20 minutes in the dark at 4°C using a PE/Dazzle™ 594 conjugated anti-human anti-PD-L1 (Cell Signaling Technology, cat. #13684) at a dilution of 1:100 in FACS buffer (composed of 1 × PBS, 2% FBS, and 2 mM EDTA). Following staining, the cells were subjected to two washes with FACS buffer and subsequently resuspended in 1×PBS prior to their analysis. The analysis of these stained cells was performed using a FACS Celesta flow cytometer (Cytek), with data processing and analysis being conducted through FlowJo software.

### 2.9 Immunoprecipitation

HCC cells underwent digestion with 0.25% trypsin and were subsequently washed twice using 1× PBS before being thoroughly lysed on ice using RIP lysis buffer, which contained 20 mM Tris (pH 7.5), 150 mM NaCl, 1% Triton X-100, a complete protease inhibitor, and 0.1 U/ml RNase inhibitor. Following this, samples were subjected to centrifugation at 12,000g for 20 minutes at 4°C, and the resulting supernatant was transferred to a fresh RNase-free EP tube. To the supernatant, Protein G Agarose beads (Thermo) were added at a volume of 50 μl per tube and incubated on a rotary shaker overnight at 4°C. The beads were then washed three times and incubated with the antibodies anti-IGF2BP1 (Cell Signaling Technology, cat. #64143), anti-c-Myc (Abcam, cat. #ab32072) at room temperature on a rotary shaker. The beads were finally collected, and RT-qPCR was employed to evaluate the enrichment efficiency.

### 2.10 Luciferase reporter assay

Initially, both wild-type and deletion variants of reporter vectors for conducting the luciferase reporter gene assay were engineered. The process involved extracting genomic DNA to amplify a 2000 bp sequence upstream (5’-UTR) of the PD-L1 gene transcription start site (TSS) by PCR, which includes a binding site for c-Myc. For the assay, cells were plated in 96-well plates and allowed to adhere overnight. These sequences were then inserted into the pGL4 luciferase vector using Bgl II and Xho I restriction enzymes, followed by ligation with T4 DNA ligase, resulting in the construct named pGL4-WT-PD-L1. After generation of c-Myc binding site mutant on pGL3-WT-PD-L1 plasmid (designated as pGL4-MT-PD-L1), subsequent transfection including wild-type or mutant reporter vectors were performed with/without overexpressing c-Myc. A scramble vector served as an internal control for the experiments. The assay was performed using dual-luciferase reporter assay kit (X-Y Biotechnology, Minhang, Shanghai, China).

### 2.11 Chromatin immunoprecipitation-PCR

ChIP experiments were conducted in alignment with the protocols provided by the EZ ChIP Chromatin Immunoprecipitation Kit (Millipore, Bedford, MA, USA). In summary, the chromatin, once crosslinked, was subjected to sonication to yield fragments ranging from 200-1,000 base pairs. The precipitation of DNA-protein complexes was achieved using anti-c-Myc and anti-IgG antibodies (Cell Signal Technology), with normal mouse immunoglobulin G (IgG) serving as a negative control. Quantification of DNA extracted from ChIP was performed through qRT-PCR, utilizing SYBR-Green dye (Applied Biosystems, Foster City, CA, USA). The sequences of the primers used are detailed in **Supplementary Table S1**.

### 2.12 RNA stability assays

HCC cells were cultured in 6-well plates until they attained an approximate density of 70%. They were then treated with Actinomycin D at a concentration of 10 μg/ml. Total RNA was isolated at time intervals of 0, 4, 8 and 12 hours, followed by the conduction of qRT-PCR to measure the levels of specific mRNA targets.

### 2.13 Fluorescence *in situ* hybridization

Oligonucleotide-modified probes for IGF2BP1, c-Myc and PD-L1 were synthesized from Sangon Biotech(Shanghai, China). Cells were washed in PBS and then treated by RNase R for 15 min to fix at 37°C. The hybridization was performed at in a dark moist chamber. After being washed twice, slices coated by cells were incubated with Alexa Fluor™ Tyramide SuperBoost™ Kits for 30 min. Slices were sealed by the parafilm containing DAPI. The *in situ* hybridization images were captured by fluorescence microscopy (OLYMPUS FV1000 confocal microscopy).

### 2.14 *In vivo* mice assay

The C57BL/6 mice (4-5 weeks) were utilized for mouse xenograft model of liver cancer by injecting 1×10^6^ mouse derived liver cancer cells (Hepa1-6) of stably transferred with IGF2BP1 shRNA and control vector. The tumor’s size, measured by caliper, was recorded for tumor volume calculation by the formula= π(a^2^b)/6. Mice were sacrificed and subjected to subsequent experiments. All the mice experiments were approved by the Committee of the Ethics of Animal Experiments of Jinhua Municipal Central Hospital.

### 2.15 Statistical analysis

All data was obtained from independent experiments. Statistical data were analyzed using SPSS 19.0 and expressed as means ± SD. Between-group was determined using Student’s t-test, and the multiple group comparison was determined using one-way analysis of variance (ANOVA). p-value less than 0.05 indicated statistical significance.

## 3 Results

### 3.1 IGF2BP1 expression was increased in HCC

**Figure 1:**
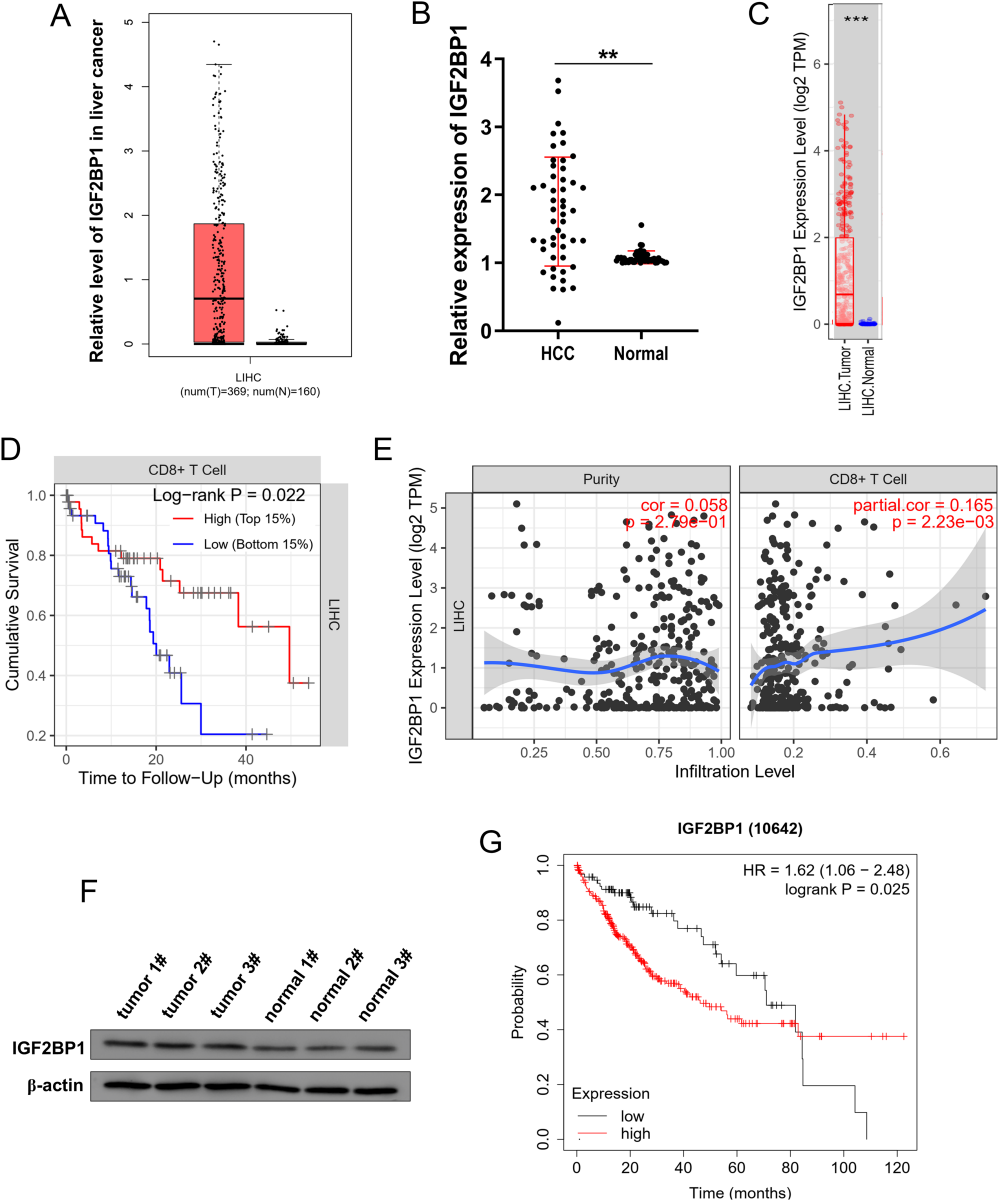
IGF2BP1 expression was increased in HCC and correlated to CD8^+^ T infiltration. **(A)** In the accessible dataset (http://gepia.cancer-pku.cn/index.html), the IGF2BP1 levels among individuals with HCC and normal controls. **(B)** IGF2BP1 levels in HCC patients compared to normal controls. **(C)** In the accessible dataset (TIMER2.0, http://timer.cistrome.org/), the IGF2BP1 levels was analyzed in HCC individuals (LIHC, Liver hepatocellular carcinoma) and normal controls. **(D, E)** The accessible dataset (TIMER2.0, http://timer.cistrome.org/) indicated the correlation within CD8^+^ T cell infiltration and survival rate in HCC cells. **(F)** Western blot analysis was employed to test the IGF2BP1 protein level in the HCC specimens and normal samples. **(G)** The survival analysis revealed that GC samples exhibiting elevated levels of IGF2BP1 were associated with a reduced survival rate. **p<0.01; ***p<0.001.

The oncogenic role of IGF2BP1 has been reported in several human cancers, however, its functions in HCC immune microenvironment and energy metabolism are still unclear. In the accessible dataset (http://gepia.cancer-pku.cn/index.html), there was a significant rise in IGF2BP1 levels among individuals with HCC (**Figure 1A**). In the samples from HCC patients, there was a significant elevation in IGF2BP1 levels compared to normal controls (**Figure 1B**). In the accessible dataset (TIMER2.0, http://timer.cistrome.org/), IGF2BP1 levels were significantly rising among individuals with HCC (**Figure 1C**). In the survival study, an increased level of CD8^+^ T cell infiltration was associated with a higher survival rate in HCC cells (**Figures 1D, E**). In the HCC specimens, a higher IGF2BP1 protein level was observed compared to normal samples (**Figure 1F**). In the survival study, an increased level of IGF2BP1 was associated with a decreased survival rate in HCC cells (**Figure 1G**). Overall, these results demonstrated that IGF2BP1 expression was increased in HCC and correlated to poor prognosis.

### 3.2 IGF2BP1 fortified oxaliplatin resistance and boosted aerobic glycolysis in HCC

**Figure 2:**
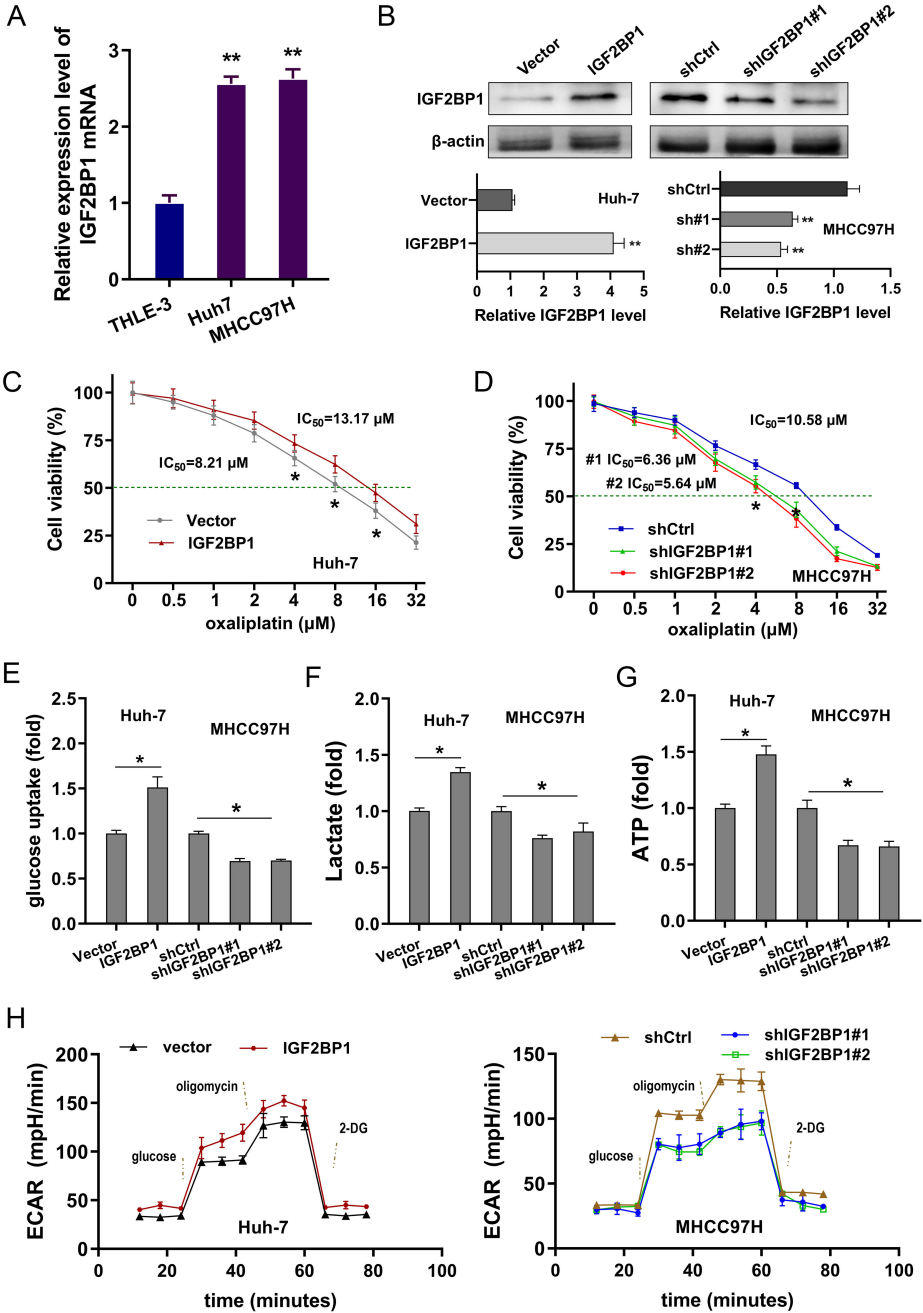
IGF2BP1 fortified oxaliplatin resistance and boosted aerobic glycolysis in HCC. **(A)** In human liver cancer cell lines (Huh7, MHCC97H) and normal human hepatic cells (THLE-3), the IGF2BP1 mRNA level was detected by RT-PCR. **(B)** Transfections to either upregulate or silence IGF2BP1 were carried out in HCC cells (Huh7, MHCC97H). The IGF2BP1 protein was detected using western blot. **(C, D)** Oxaliplatin sensitivity by CCK-8 assay showed the IC_50_ value (Oxaliplatin concentration causing 50% decrease in absorbance compared with the control) of HCC cells with IGF2BP1 upregulation or silencing. **(E-G)** The level of glucose uptake **(E)**, lactate production **(F)** and ATP **(G)** were detected in the HCC cells with IGF2BP1 upregulation or silencing. **(H)** The examination of metabolic flux for extracellular acidification rate (ECAR) was examined in overexpressing IGF2BP1 or silencing IGF2BP1 in HCC cells. All experiment data were independently repeated at least triplicate and normalized to respective controls. *p<0.05, **p<0.01.

Given that our previous findings showed the oncogenic role of IGF2BP1 on HCC, the following assays were performed to test the specific biologic role. Firstly, the IGF2BP1 mRNA level was increased in human liver cancer cell lines (Huh7, MHCC97H) comparing to normal human hepatic cells (THLE-3) (**Figure 2A**). Subsequently, transfections to either upregulate or silence IGF2BP1 were carried out in HCC cells (**Figure 2B**). Firstly, results found that oxaliplatin sensitivity analysis revealed that overexpressing IGF2BP1 increased the IC_50_ value (the concentration of oxaliplatin needed to achieve a 50% reduction in absorbance relative to the control) of HCC cells, whereas IGF2BP1 silencing reduced the IC_50_ value (**Figures 2C, D**). In cancer cells, changes in glucose metabolism meet the needs of self-proliferation, angiogenesis, metastasis and also affect the immune escape. Therefore, the research investigated the role of IGF2BP1 on HCC glucose metabolism, specifically aerobic glycolysis. To determine the state of glycolysis in HCC, the analysis of glucose uptake, lactate production and ATP was performed. Results illustrated that overexpressing IGF2BP1 enhanced the level of glucose uptake (**Figure 2E**), lactate production (**Figure 2F**) and ATP (**Figure 2G**). While, the suppression of IGF2BP1 reduced the glucose uptake, lactate production and ATP. The examination of metabolic flux for extracellular acidification rate (ECAR) demonstrated that overexpressing IGF2BP1 increased the rate of glycolysis in HCC cells (**Figure 2H**), whereas suppressing IGF2BP1 decreased the glycolysis rate. Taken together, these data concluded that IGF2BP1 fortified oxaliplatin resistance and boosted aerobic glycolysis in HCC.

### 3.3 IGF2BP1 exacerbated CD8^+^ T cells mediated immune escape in HCC

**Figure 3:**
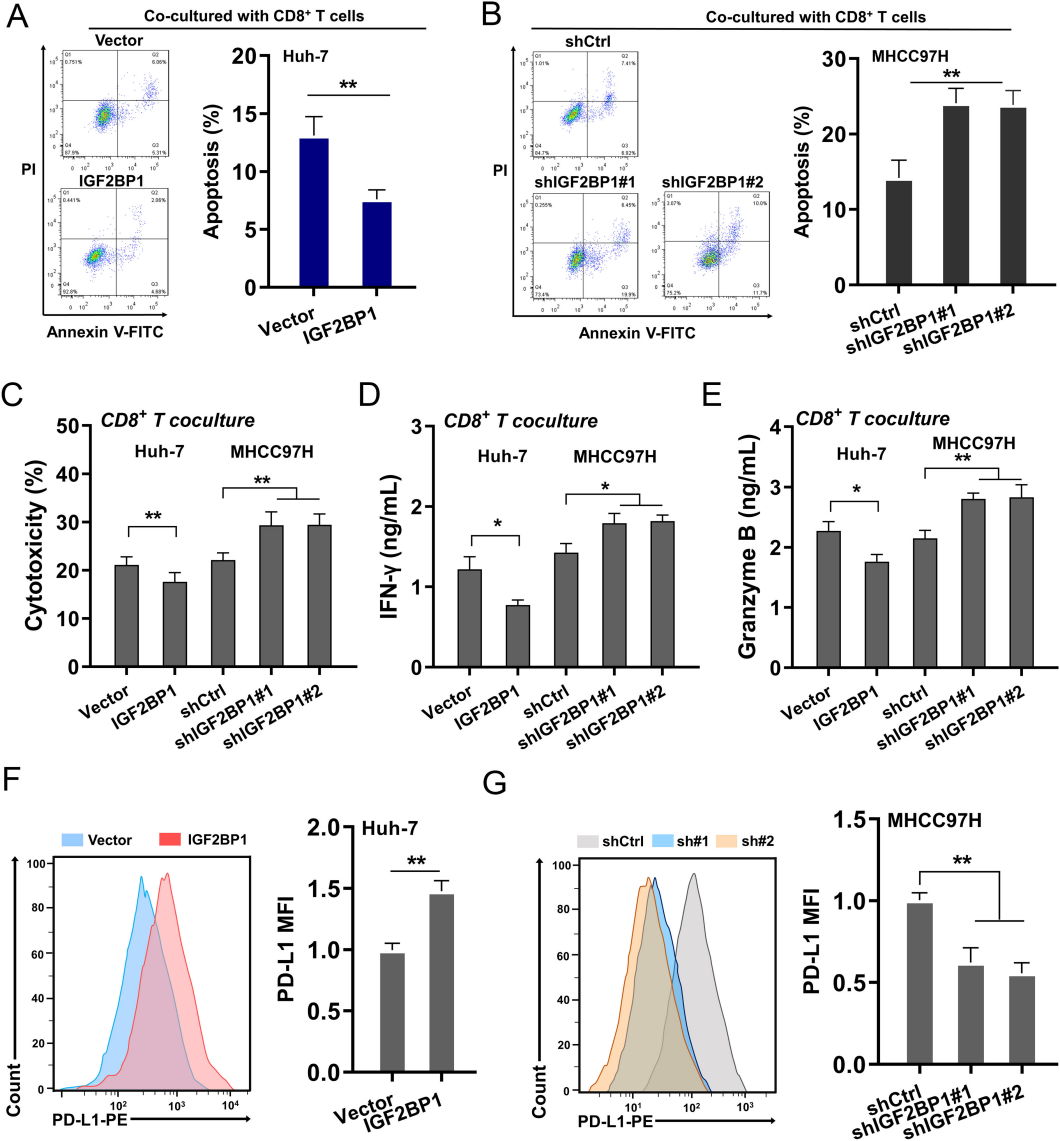
IGF2BP1 exacerbated CD8^+^ T cells mediated immune escape in HCC. **(A, B)** Apoptosis in HCC cells within the coculture system was assessed by flow cytometry. HCC cells were transfected with IGF2BP1 overexpression and silencing. **(C)** The cytotoxicity was measured by LDH release analysis that secreted from cocultured CD8^+^ T cells. **(D, E)** The cytokines secreted by CD8^+^ T cells were tested by ELISA kit, including **(D)** IFN-γ secretion, **(E)** granzyme **(F, G)** Flow cytometry was utilized to detect the expression of PD-L1 on the surface of HCC cells. All experiment data were independently repeated at least triplicate and normalized to respective controls. *p<0.05; **p<0.01.

Our preliminary results confirmed the impact of IGF2BP1 on the clinical prognosis of HCC patients and the CD8^+^ T infiltration immune microenvironment, indicating that IGF2BP1 could potentially control the anti-tumor immunity of CD8^+^ T cells within the HCC microenvironment. To examine the role of IGF2BP1 in enhancing the cytotoxic activity of CD8^+^ T cells against HCC cells, a coculture system comprising activated CD8^+^ T cells and HCC cells was established. Apoptosis in HCC cells within the coculture system was assessed, and findings demonstrated that elevated IGF2BP1 expression decreased HCC cell apoptosis, while silencing of IGF2BP1 expedited this apoptotic process (**Figures 3A, B**). To explore the impact of IGF2BP1 on the antitumor response mediated by CD8^+^ T cells, the characteristics of CD8^+^ T cells in the co-culture system were monitored. The cytotoxicity assay of T cells, measured through LDH release, revealed that CD8^+^ T cells exhibited significantly reduced cytotoxic activity against HCC cells transfected with high-expression IGF2BP1 compared to those transfected with control vectors (**Figure 3C**). The findings demonstrated that overexpressing IGF2BP1 compromised the antitumor immune capabilities of CD8^+^ T cells, whereas knockdown of IGF2BP1 led to a decline in their antitumor immune functions, including IFN-γ levels (**Figure 3D**) and granzyme B (**Figure 3E**). Furthermore, the expression of PD-L1 on the surface of HCC cells was measured, revealing that overexpression of IGF2BP1 enhanced the expression of PD-L1 on the cellular surface of HCC cells, whereas silencing IGF2BP1 led to a reduction in PD-L1 surface expression (**Figures 3F, G**). Collectively, the data from this study revealed IGF2BP1 exacerbated CD8^+^ T cells mediated immune escape in HCC.

### 3.4 IGF2BP1 enhanced the stability of c-Myc mRNA

**Figure 4:**
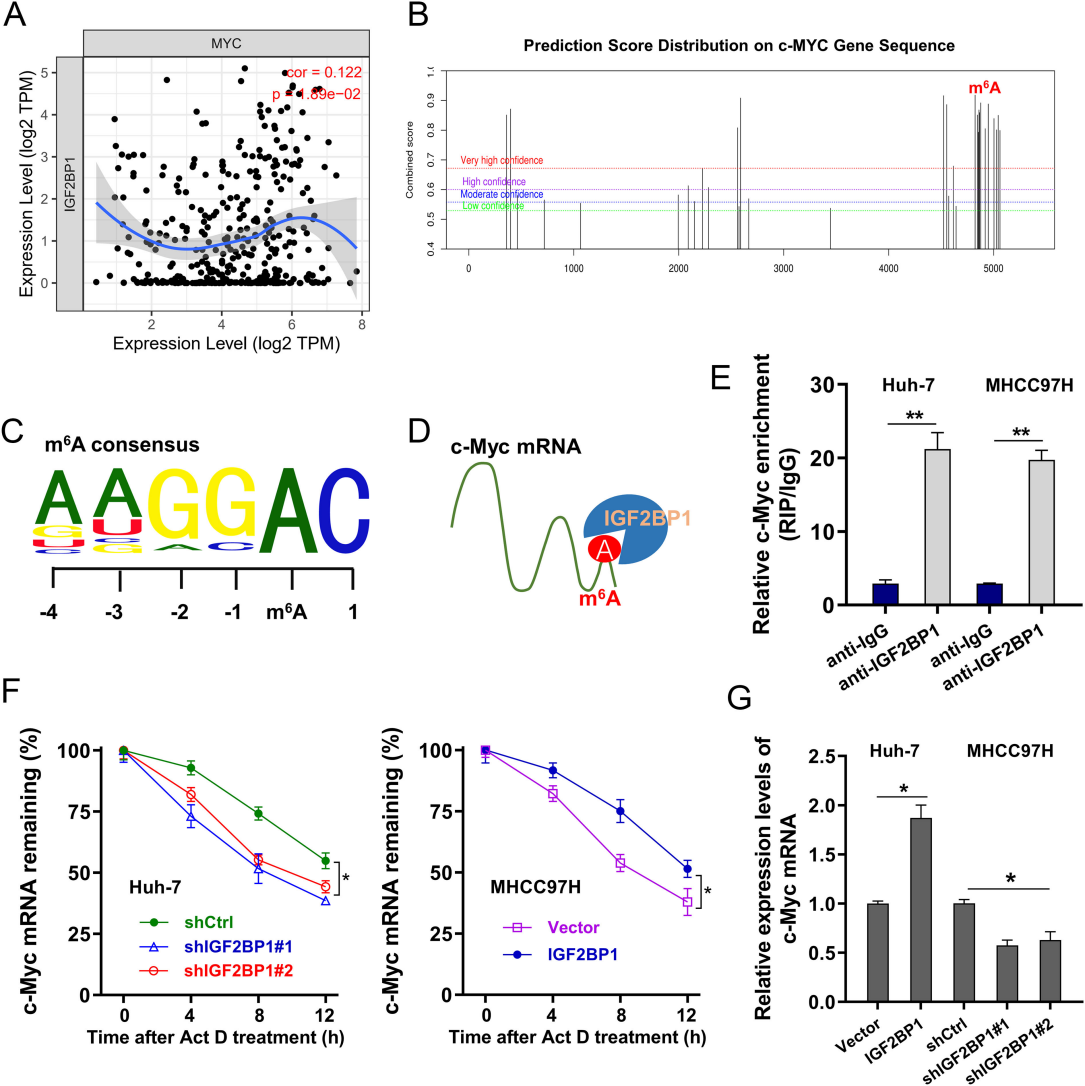
IGF2BP1 enhanced the stability of c-Myc mRNA. **(A)** The public DataBase (TIMER 2.0, http://timer.cistrome.org/) illustrated the positive correlation within IGF2BP1 and c-Myc. **(B)** The SRAMP (http://www.cuilab.cn/sramp) illustrated the forecasted significant m^6^A sites on the mRNA of c-Myc. **(C)** The m^6^A consensus on the c-Myc mRNA was GGAC. **(D)** Schematic diagram highlighted the binding of m^6^A reader IGF2BP1 on m^6^A modified c-Myc mRNA. **(E)** RIP-PCR indicated the precipitated c-Myc mRNA integrated by IGF2BP1 antibody. **(F)** The RNA decay assay demonstrated that the level of remaining c-Myc mRNA in HCC cells treated with Act **(D, G)** RT-PCR evidenced the c-Myc mRNA in HCC cells transfected with IGF2BP1 overexpression or silencing. All experiment data were independently repeated at least triplicate and normalized to respective controls. *p<0.05; **p<0.01.

The public DataBase showed that IGF2BP1 was positively correlated to c-Myc (**Figure 4A**). In the SRAMP (http://www.cuilab.cn/sramp), the forecasted outcomes revealed the presence of significant m^6^A sites on the mRNA of c-Myc (**Figure 4B**). The m^6^A consensus on the c-Myc mRNA was GGAC (**Figure 4C**). The m^6^A modified c-Myc mRNA could be combined by m^6^A reader IGF2BP1 (**Figure 4D**). RIP-PCR indicated that m^6^A reader IGF2BP1 could noticeably interacted with c-Myc mRNA in the HCC transcript (**Figure 4E**). To verify how IGF2BP1 determine the fate of c-Myc mRNA, the transcript stability assay (RNA decay assay) was performed and results indicated that IGF2BP1 silencing reduced the c-Myc mRNA remaining level upon Act D treatment, and the IGF2BP1 overexpression up-regulated the c-Myc mRNA remaining level, suggesting the reinforcement of IGF2BP1 on c-Myc mRNA stability (**Figure 4F**). Moreover, the IGF2BP1 overexpression increased the c-Myc mRNA level and IGF2BP1 silencing decreased the c-Myc mRNA level (**Figure 4G**). To generalize, the outcomes of this investigation indicated IGF2BP1 enhanced the stability of c-Myc mRNA.

### 3.5 c-Myc bolstered the transcriptional activity of PD-L1 in HCC

**Figure 5:**
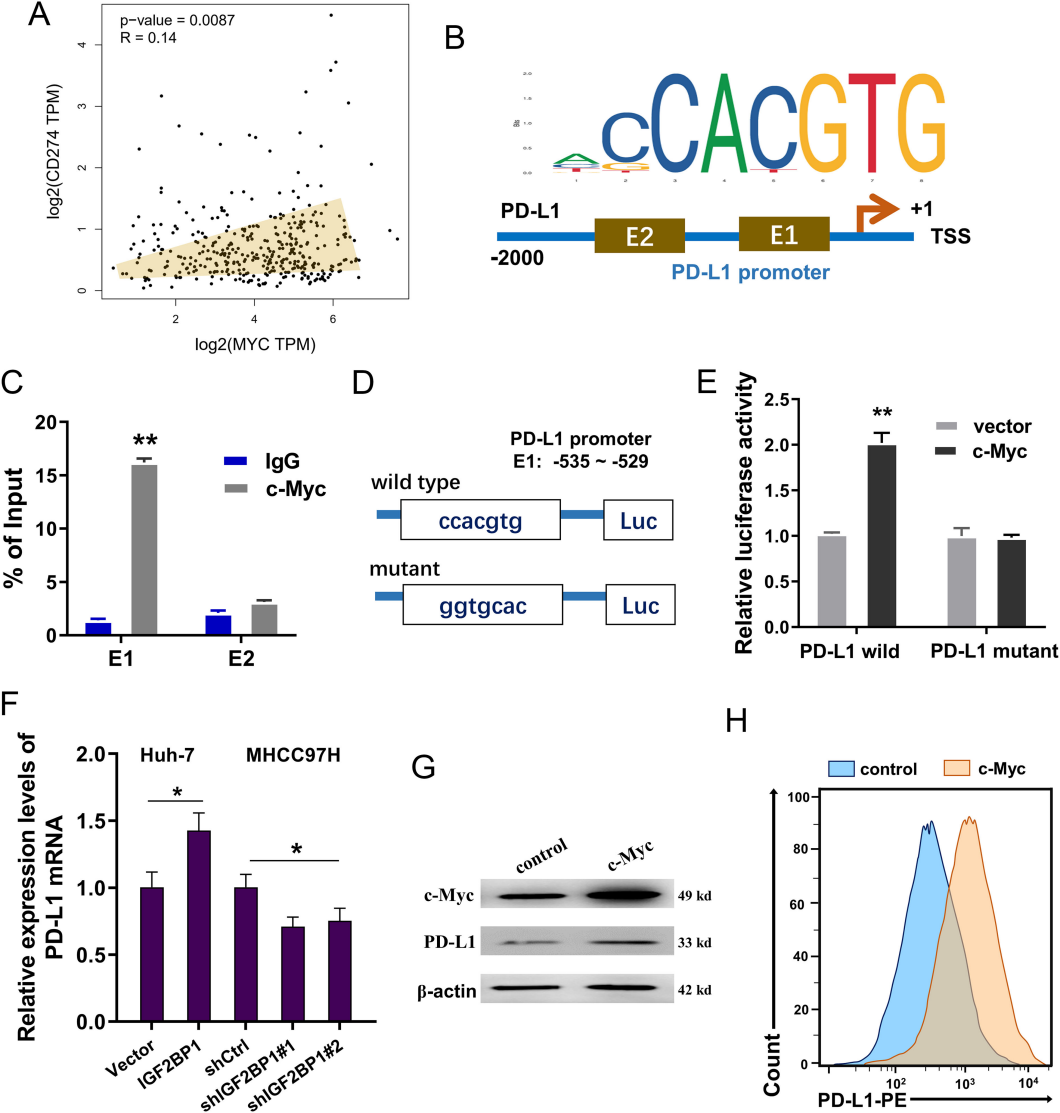
c-Myc bolstered the transcriptional activity of PD-L1 in HCC. **(A)** In HCC samples, the correlation within c-Myc and PD-L1 was positive (GEPIA, http://gepia.cancer-pku.cn/index.html). **(B)** Using JASPAR (http://jaspar.genereg.net/) and RegRNA (http://regrna2.mbc.nctu.edu.tw/detection.html), two likely binding sites (E1: –535~-529; E2: -1307~ -1301) were identified on PD-L1 transcript. **(C)** In Huh-7 cells, ChIP-PCR was conducted to identify the enrichment of E1/E2 putative binding sequences with the c-Myc antibody. IgG served as the negative control. **(D)** Luciferase reporter constructs of both wild type and mutant sequences for the PD-L1 promoter region (-535~-529) were constructed. **(E)** The luciferase reporter assay demonstrated the luciferase activities after the vectors (wild type or mutant) and c-Myc. **(F)** RT-PCR showed the PD-L1 mRNA in HCC cells transfected with IGF2BP1 overexpression or silencing. **(G)** Western blot assay indicated the c-Myc protein and PD-L1 protein levels in Huh-7 cells transfected with c-Myc overexpression. **(H)** Flow cytometry was utilized to detect the expression of PD-L1 on the surface of HCC cells. All experiment data were independently repeated at least triplicate and normalized to respective controls. *p<0.05; **p<0.01.

Our previous data indicated that IGF2BP1 and c-Myc collaboratively promoted the surface PD-L1 expression on HCC cells. Therefore, the next assays will aim to closely scrutinize their interaction within PD-L1. In HCC samples, the correlation within c-Myc and PD-L1 was positive (**Figure 5A**). Using JASPAR (http://jaspar.genereg.net/) and RegRNA (http://regrna2.mbc.nctu.edu.tw/detection.html), two likely binding sites were identified on PD-L1 transcript (**Figure 5B**). Through conducting ChIP-PCR aimed at these sites, it was found that the initial site (-535~-529) could efficiently bind with the c-Myc antibody (**Figure 5C**). Constructs of both wild type and mutant sequences for the PD-L1 promoter region (-535~-529) were constructed (**Figure 5D**). The luciferase reporter assay demonstrated that the c-Myc could bind to the wild type region, implying an activation of PD-L1 transcription mediated by c-Myc (**Figure 5E**). Moreover, the IGF2BP1 overexpression increased the PD-L1 mRNA level and IGF2BP1 silencing decreased the PD-L1 mRNA level (**Figure 5F**). The c-Myc overexpression could promote the PD-L1 protein level (**Figure 5G**). Furthermore, the expression of PD-L1 on the surface of HCC cells was augmented by c-Myc overexpression (**Figure 5H**). Collectively, the data from this study revealed that c-Myc bolstered the transcriptional activity of PD-L1 in HCC.

### 3.6 IGF2BP1 targeted c-Myc/PD-L1 to accelerate aerobic glycolysis and immune escape of HCC

**Figure 6:**
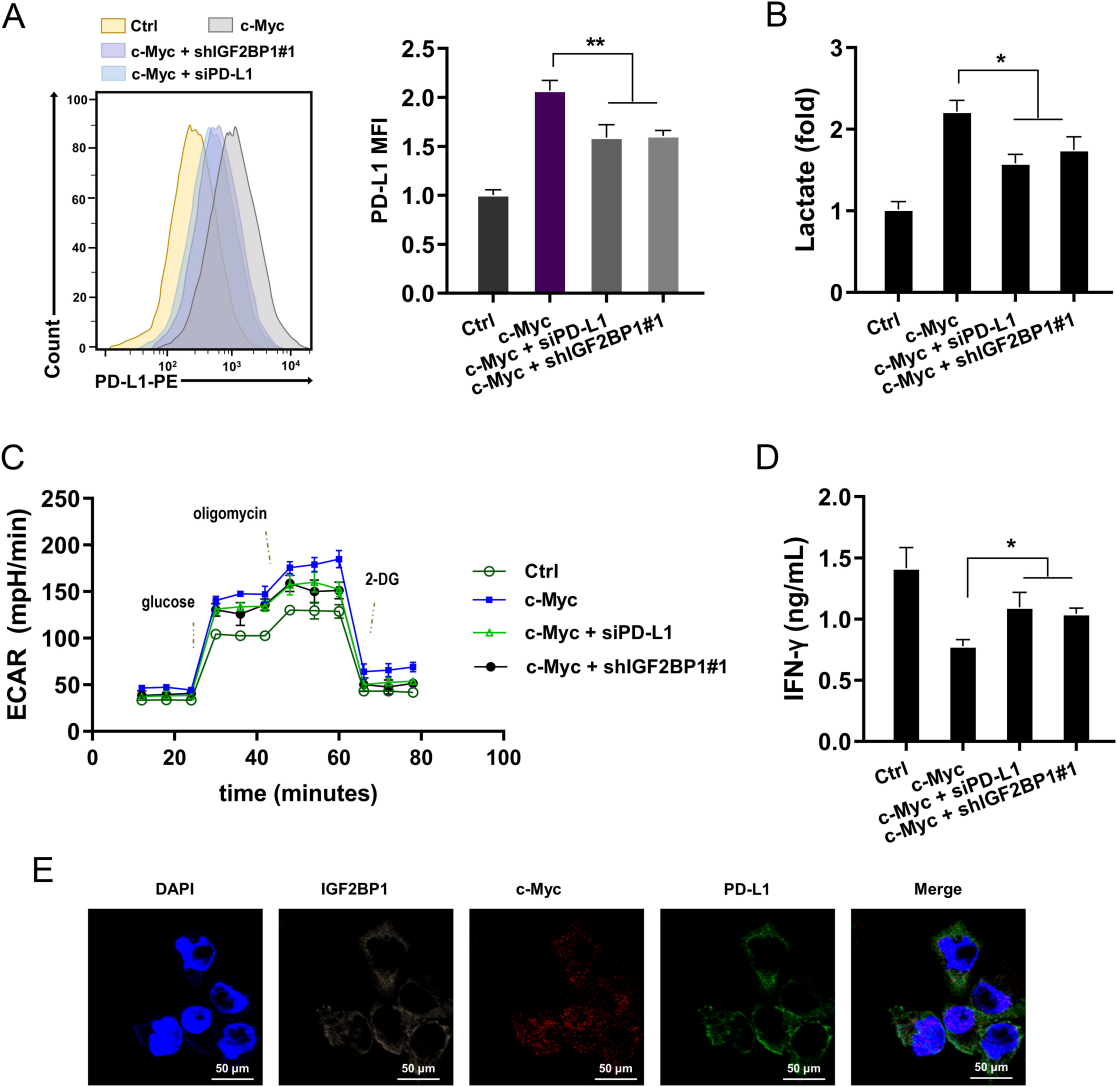
IGF2BP1 targeted c-Myc/PD-L1 to accelerate aerobic glycolysis and immune escape of HCC. **(A)** Flow cytometry was utilized to detect the expression of PD-L1 on the surface of HCC cells (MHCC97H). **(B)** Lactate analysis was performed in HCC cells (MHCC97H). **(C)** The examination of metabolic flux for extracellular acidification rate (ECAR) was examined in overexpressing c-Myc or silencing of IGF2BP1/PD-L1 in HCC cells. **(D)** The lactate was analyzed in HCC cells. **(E)** The subcellular location analysis in HCC cells (MHCC97H) using probes of IGF2BP1, c-Myc and PD-L1. All experiment data were independently repeated at least triplicate and normalized to respective controls. *p<0.05, **p<0.01.

The following assays were performed to verify the role of IGF2BP1 on HCC aerobic glycolysis and immune escape by targeting c-Myc/PD-L1 axis. Firstly, the expression of PD-L1 on the surface of HCC cells (MHCC97H) was measured, revealing that overexpression of c-Myc enhanced the expression of PD-L1 on the cellular surface of HCC cells, whereas IGF2BP1 silencing and PD-L1 silencing both reduced the PD-L1 surface expression (**Figure 6A**). The lactate and ECAR analysis revealed that overexpression of c-Myc promoted the lactate and ECAR level, and IGF2BP1/PD-L1 silencing both inhibited them (**Figures 6B, C**). For the antitumor immune functions of cocultured CD8^+^ T cells, the IFN-γ level was calculated. Data indicated that overexpression of c-Myc repressed the lactate and ECAR level, and IGF2BP1/PD-L1 silencing both up-regulated them (**Figure 6D**). The subcellular location analysis found that IGF2BP1 could co-located with c-Myc/PD-L1 in HCC (**Figure 6E**). Collectively, the data from this study revealed that IGF2BP1 targeted c-Myc/PD-L1 to accelerate aerobic glycolysis and immune escape of HCC.

### 3.7 IGF2BP1 silencing repressed the PD-L1 level *in vivo*

**Figure 7:**
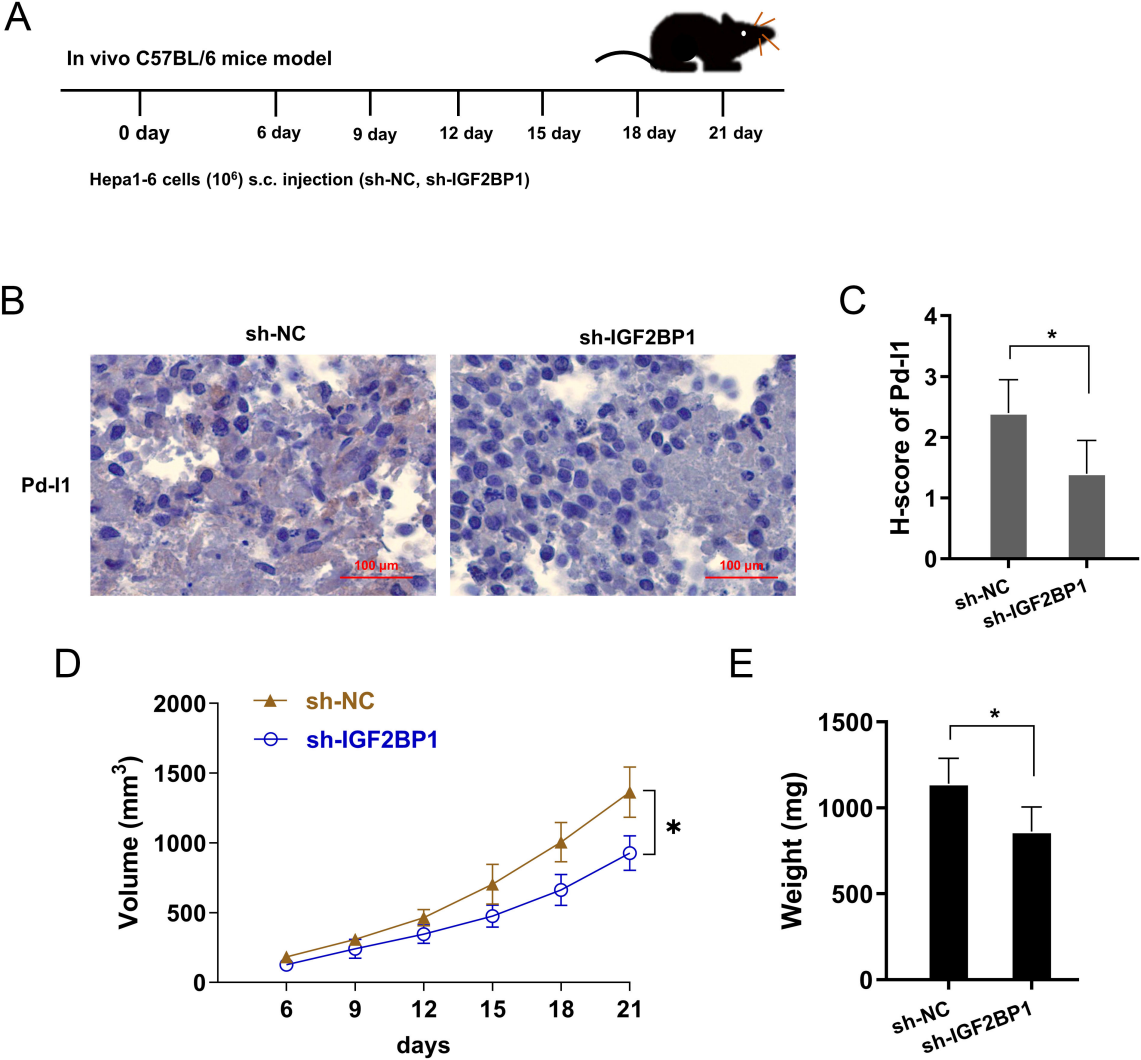
IGF2BP1 silencing repressed the PD-L1 level *in vivo*. **(A)** The xenograft mice assay was performed using the mouse derived liver cancer cells (Hepa1-6) of stably transferred with IGF2BP1 shRNA#1 (sh-IGF2BP1) and control vector (sh-NC). **(B, C)** Immumohistochemical staining for PD-L1 in tumor tissue, and the H-score analysis. **(D, E)** The tumor volume and weight analysis revealed the volume **(D)** and weight **(E)** with IGF2BP1 silencing and control. *p<0.05.

To investigate the role of IGF2BP1 on PD-L1 level *in vivo*, the xenograft mice assay was performed using the mouse derived liver cancer cells (Hepa1-6) of stably transferred with IGF2BP1 shRNA#1 (sh-IGF2BP1) and control vector (sh-NC) (**Figure 7A**). In the tumor tissue, the IGF2BP1 silencing reduced the PD-L1 level (**Figures 7B, C**). The tumor volume and weight analysis revealed that IGF2BP1 silencing inhibited the volume (**Figure 7D**) and weight (**Figure 7E**). Collectively, the data from this study revealed that IGF2BP1 silencing repressed the PD-L1 level *in vivo*.

## 4 Discussion

Recent studies on hepatocellular carcinoma (HCC) have highlighted the critical role of N^6^-methyladenosine (m^6^A) modifications in its energy metabolism and immune microenvironment (14–16). m^6^A modifications, known as “epitranscriptomic” changes, have been implicated in the post-transcriptional regulation of numerous gene expression, influencing series of tumor processes. Emerging evidence also suggests that specific m^6^A ‘readers’, such as IGF2BP1, can modulate the stability and translation of target mRNAs, thereby affecting tumor growth and immune escape in HCC (17, 18). Understanding the tumor microenvironment of HCC is critical for developing effective immunotherapeutic approaches, as it plays a significant role in immune escape and therapy resistance.

The interdependence of aerobic glycolysis and immune escape in cancer progression is underscored by the role of glycolytic metabolites in modulating key immune checkpoints, thereby directly influencing the immune system’s ability to recognize and attack tumor cells (19). Immunotherapy for HCC represents a promising avenue for treatment, aiming to enhance the immune system’s ability to recognize and eliminate cancer cells (20). The phenomenon of aerobic glycolysis, often referred to as the Warburg effect in cancer cells, has been implicated in creating an immunosuppressive microenvironment that facilitates immune escape (21). Studies indicate that the metabolic reprogramming of cancer cells to aerobic glycolysis contributes to the suppression of anti-tumor immune responses, thereby establishing a causal pathway that aids in immune escape (22).

**Figure 8:**
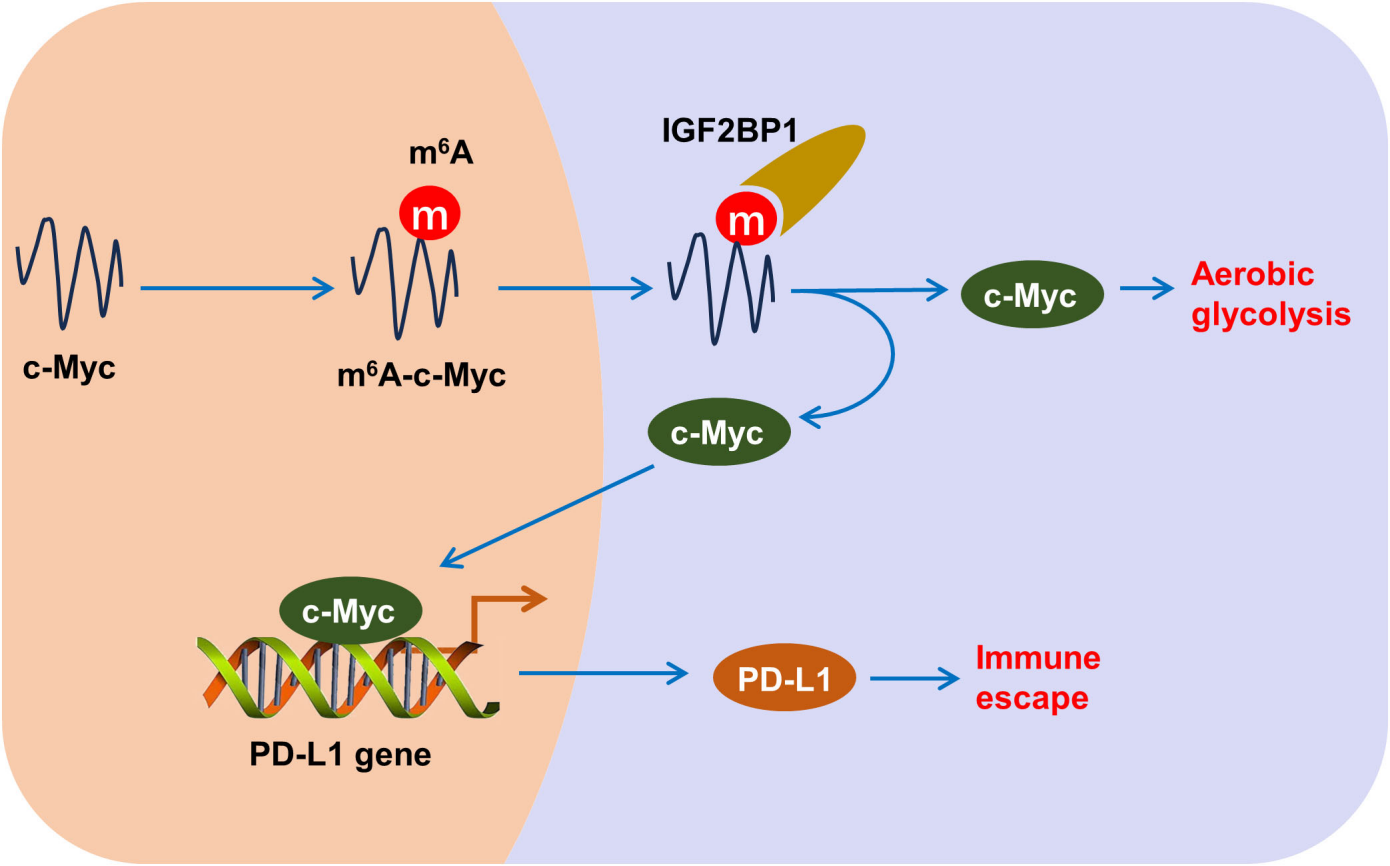
m^6^A reader IGF2BP1 induces aerobic glycolysis and immune escape of HCC by targeting c-Myc/PD-L1.

Here, this research aimed to test the function of m^6^A reader IGF2BP1 on HCC aerobic glycolysis and immune escape (**Figure 8**). Results indicated that elevated IGF2BP1 expression was associated with poor prognosis and lack of CD8^+^ T cell infiltration in HCC patients. Functionally, IGF2BP1 emerged as an oncogenic factor that accelerated HCC aerobic glycolysis (glucose uptake, lactate generation and ECAR), and repressed the activated CD8^+^ T cell-mediated killing effect (cytotoxicity, IFN-γ and granzyme B) and apoptosis. Specifically, this study found that the m^6^A reader IGF2BP1 facilitated immune escape in HCC cells, which was evidenced by reduced lactate dehydrogenase (LDH) release, Granzyme B and IFN-γ production, indicating a suppressed cytotoxic T cell response. Besides, IGF2BP1 facilitated the aerobic glycolysis of HCC by enhancing c-Myc mRNA’s stability. Additionally, the finding observed decreased apoptosis of HCC cells when co-cultured with CD8^+^ T cells, further suggesting that IGF2BP1 may contribute to the immune escape by modulating the HCC tumor microenvironment.

IGF2BP1 has been implicated in the progression and immune escape of other cancers as well. For instance, in gastric cancer, IGF2BP1 overexpression augments the proliferation of co-cultured gastric cancer cells, and mitigates the CD8^+^ T cells mediated anti-tumor response, including IFN-γ secretion, surface PD-L1 level, and cytotoxicity of CD8^+^ T cells (23). In colon cancer, upregulation of IGF2BP1 suppresses the CD8^+^ T-cells mediated antitumor immunity through reducing the tumor cytotoxicity and enhancing the target PD-L1 mRNA stability. The interaction between PD-L1 and immune cells plays a pivotal role in tumor immune escape in HCC. PD-L1 is known to bind to PD-1 on the surface of T cells, resulting in the inhibition of T cell activation and proliferation. Here, our findings also confirmed that IGF2BP1 induces aerobic glycolysis and immune escape of HCC. Moreover, the programmed death ligand 1 (PD-L1) mRNA had a remarkable m^6^A modified site on 3’-UTR genomic. Therefore, PD-L1 acted as of IGF2BP1 through the m^6^A modified site binding. Then, c-Myc acted as the transcription factor and could strengthen the transcription of PD-L1 gene. In conclusion, IGF2BP1 fortified the HCC aerobic glycolysis and immune escape by targeting c-Myc/PD-L1 manner. In human cancer, c-Myc is a classical transcription factor that promotes tumorigenesis (24–26). Here, the elevated c-Myc then acts as a transcriptional activator, notably binding to the promoter region of the PD-L1 gene, a critical immune checkpoint ligand. This interaction augments PD-L1 transcription, thereby bolstering its protein levels on the surface of HCC cells. The surge in PD-L1 serves as a mechanism for tumor cells to evade immune destruction by engaging the PD-1 receptor on T cells, leading to the suppression of T cell-mediated cytotoxicity.

In conclusion, our research uncovered a regulation wherein IGF2BP1 enhances aerobic glycolysis and immune escape in HCC by stabilizing PD-L1 mRNA, leading to reduced cytotoxicity of CD8^+^ T cells against HCC cells. Taken together, this study unveiled that IGF2BP1 functioned as an oncogenic element that deteriorated the HCC cells’ aerobic glycolysis and immune escape from lymphocyte killing effect. These findings highlight the potential of m^6^A regulators IGF2BP1 by enhancing PD-L1 mRNA stability via targeting c-Myc/PD-L1 manner. This cascade reaction is a key mechanism by which IGF2BP1 helps HCC cells avoid immune surveillance.

## Data Availability

The original contributions presented in the study are included in the article/**Supplementary Material**. Further inquiries can be directed to the corresponding author.
