# Supplementary material for: IGF2BP1 accelerates the aerobic glycolysis to boost its immune escape in hepatocellular carcinoma microenvironment
Source: Front Immunol. 2024 Nov 13;15:1480834. doi: 10.3389/fimmu.2024.1480834 (PMC11599169; doi:10.3389/fimmu.2024.1480834)
Supplement: Supplementary file 1 [file Table1.docx]

**supplementary table S1**. qRT-PCR primers sequences and shRNA sequences.

|  | Sequences |
| --- | --- |
| IGF2BP1 | F, 5’- GGCCATCGAGAATTGTTGCAG-3’  R, 5’- CCAGGGATCAGGTGAGACTG-3’ |
| PD-L1 | F, 5’- GGACAAGCAGTGACCATCAAG-3’  R, 5’- CCCAGAATTACCAAGTGAGTCCT-3’ |
| sh-IGF2BP1-1 | 5’- CTCCGCTTGTAAGATGATCTT-3’ |
| sh-IGF2BP1-2 | 5’- CGACCAAGTCATTGTTAAGAT-3’ |
| GAPDH | F, 5’-CAGGAGGCATTGCTGATGAT-3’  R, 5’-GAAGGCTGGGGCTCATTT-3’ |
